# Molecular genetics solutions to grand challenges in Entomology

**DOI:** 10.3389/finsc.2022.999165

**Published:** 2022-09-20

**Authors:** Subba Reddy Palli

**Affiliations:** Department of Entomology, University of Kentucky, Lexington, KY, United States

**Keywords:** public health, natural resources, invasive species, food security, genome sequencing

## Grand challenges in Entomology

The Entomological Society of America (ESA) started an initiative to bring together a coalition of experts to identify grand challenges in Entomology and find sustainable solutions to these challenges (https://entomologychallenges.org/). This coalition identified three major grand challenges; public health, feed the world and invasive species. These three grand challenges identified are indeed huge problems faced all over the world at this time. In this grand challenge article to the Insect Molecular Genetics section of Frontiers of Insect Science journal, I will articulate how Insect Molecular Genetics covered by this section could contribute to developing solutions to grand challenges in Entomology.

1. Public health: one of the grand challenges of entomologists is the threat of insects that cause public health problems. Vector-borne diseases caused by parasites, bacteria and viruses account for 17% of infectious diseases and 700,000 deaths worldwide (https://www.who.int/news-room/fact-sheets/detail/vector-borne-diseases). Malaria parasites transmitted by Anopheline mosquitoes are responsible for more than half of the deaths resulting from infectious diseases. Dengue viruses transmitted by *Aedes* mosquitoes are responsible for 40,000 deaths in 129 countries. Mosquitoes also transmit viruses that cause many diseases including Chikungunya fever, Dengue fever, Lymphatic filariasis, Rift Valley fever, Yellow Fever, Zika and West Nile fever (1–3).
Other insects such as fleas, blackflies, lice, sandflies, tsetse flies and triatome bugs transmit parasites and pathogens that cause human and animal diseases. Other arthropods such as ticks, and mites also pose a serious threat to human and animal health by acting as vectors of pathogens that cause infectious diseases. Crimean-Congo hemorrhagic fever, Lyme disease, Relapsing fever, Rickettsial diseases, Tick-borne encephalitis and Tularaemia are important diseases caused by bacteria and viruses transmitted by ticks (4). An increase in international travel, global warming and insecticide resistance are contributing to a rapid rise in infectious disease incidence. The jumping of zoonotic viruses from animals to humans leading to pandemics is also a major concern. Zika virus transmitted by *Aedes aegypti* mosquitoes is an example of an insect-transmitted virus jumping from animals to humans (5).
Household pests, including bed bugs, cockroaches, and houseflies, cause major health problems, including allergies, mental health, and nuisance. The infestations by household pests are on the rise again due to similar reasons listed above for the increase in the incidence of disease vectors. Most of the vector-borne diseases are preventable through vector control and the use of protective measures such as repellents, insecticide-treated bed nets and clothing. Insect molecular genetics could help in developing new vector control methods, insecticides, and repellents to combat emerging vector-borne diseases.
2. Feed the world and save the natural resources: As the world population grows, there is a continuous demand to produce more food on the limited farmland. The challenge is to increase food production for the growing population without expanding agricultural land to preserve natural resources on planet earth. Nearly 50% of food produced is lost to pests, diseases and weeds that affect crops during the preharvest stage and to pests, pathogens and rodents during the post-harvest stage. Protection of crops, trees and their products from insect pests could result in a significant boost in food production, storage, and distribution. However, this is always not possible due to challenges posed by the non-target effects of toxic insecticides on beneficial insects, animals and humans resulting in the decline of bees that pollinate crops and deteriorating human and animal health. Recent reports on the effect of imidacloprid insecticides on honeybees and the destruction of food crops by fall armyworm in Africa and Asia leading to food security issues are examples of issues arising out of non-target effects and resistance development problems of insecticides (6). These issues are making controlling insect pests that destroy crops, trees and their products a grand challenge threatening food security. Novel and target-specific insecticides affecting non-neuronal targets in insects are an urgent need that could be addressed by research in insect molecular genetics.
3. Invasive species: Insects introduced to one region of the world from the other regions threaten health of humans, animals, natural resources, and crops and are therefore considered a grand challenge facing humanity (7, 8). Due to internationalization, increased global trade and tourism, there is a continuous threat of insects being transported from one region of the world to the other region. Some of these insects establish themselves as major pests that threaten global food security and the preservation of natural resources. Many invasive insects such as Emerald ash borer, Asian longhorn spongy, spongy moth, brown marmorated stink bug, etc. already destroyed many crops and trees in forests, parks, recreation facilities and backyards. Advanced methods to detect and destroy invasive pests to limit their establishment and spread are urgently needed. Insect molecular genetics could help in developing such methods.

## Molecular genetics solutions

1. Sequencing and functional genomics: Multiple projects underway to sequence genomes, transcriptomes, and proteomes of insects, along with recent advances in functional genomics methods, including RNA interference and genome editing, will help in better understanding of insect biology paving the way to find novel target sites and methods to control them. The first whole-genome sequencing of an insect, the fruit fly was completed in 2000 and closely flowed by completing the genome sequence of a disease vector, *Anopheles gambiae* in 2002. Since then, sequencing of hundreds of whole genomes and thousands of transcriptomes of insects were completed. Insect genome sequencing initiatives such as i5K (sequencing of 5000 arthropod genomes), 1KITE (1K Insect Transcriptome Evolution), the earth biogenome and other such initiatives are playing important roles in sequencing insect genomes and transcriptomes (9–11). Recent advances in proteomics, single-cell sequencing and multiomics approaches will help in identifying genes, RNA and proteins in most of the economically and ecologically important insect species. Functional genomics methods, including RNA interference, genome editing and other methods, will help to learn the functions of genes, RNAs and proteins identified by sequencing efforts.
Integration and analysis of data from sequencing projects is a major challenge. Perhaps, artificial intelligence might help in the analysis and interpretation of data, especially those from multiomics efforts. Epigenetics, including modification of DNA, RNA and proteins as well as microRNAs in the regulation of genome expression will lead to a deeper understanding of the biology of insects. Advanced methods such as interactomes looking at protein:protein and proteins:RNA interaction and metabolomics studying changes in lipids, carbohydrates and other metabolites will help to understand intricate pathways that govern developmental, reproductive and physiological processes in insects. Recent advances in single-cell sequencing methods are revealing cell-specific changes at unprecedented levels (12).
2. Surveillance, Speciation, and Screening for pathogens: Identification and eradication of insects that transmit human, animal, and plant diseases, especially those coming through transportation, is important for preventing the spread of vector-borne diseases. Detection of pathogens that jump from animals to humans also requires efficient methods for their detection. Detection of nucleic acids and proteins produced by insects and pathogens by various methods, including hybridization, polymerase chain reaction and antibody methods, has been used for many years. Novel methods are also being developed for use in the surveillance of vectors and screening for pathogens they transmit (13).
3. New target sites and novel management methods: Recent advances in genomics and functional genomics approaches and their applications to study pest biology could help in the identification of new target sites for the development of pest management methods that are target specific. Recent development of RNAi-based methods to manage beetle pests such as corn rootworm and Colorado potato beetle are good examples of the contribution of new technology to the development of novel pest management methods (14). Significant advances have been made in the application of genome editing approaches such as CRISPR/Cas9 for developing methods to control disease vectors such as *Aedes aegypti* (15). The potential for the development of pest and disease vector control methods based on recent developments in molecular genetics approaches is enormous.
4. Systems Biology of insecticide resistance development: There has been continuous competition between insects and humans for resources on this planet. As humans discovered and developed better synthetic insecticides for controlling pests and disease vectors, insects found ways to resist them. Insects developed resistance to almost all synthetic insecticides deployed to destroy them. Research aimed at understanding insecticide resistance led to the discovery of multiple approaches such as target site mutations, increased metabolism, avoidance behavior and decreased penetration of insecticides employed by insects to resist insecticides. Recent advances in molecular methods contributed to a compressive understanding on mechanisms of insecticide resistance (16). For example, genes coding for enzymes such as P450 monooxygenases that contribute to enhanced metabolism of insecticides in resistant insects have been identified in multiple insect pests. In addition, transcription factors such as CncC that are responsible for the increase in expression of insecticide metabolizing enzymes in resistant insects have been identified and the mechanisms of their action were discovered (17).

## Conclusions and perspective

As explained above, molecular genetics will play an important role in addressing grand challenges in entomology. As new methods are developed and applied to address grand challenges in entomology, there will be numerous discoveries worthy of publication. The Molecular Genetics section of Frontiers in Insect Science will be interested in publishing your manuscripts describing your research in genetics, epigenetics, genomics, transcriptomics, RNAi (RNA interference), genome editing, proteomics, metabolomics diagnostics, insecticide resistance and other related areas.

## Author contributions

SP conceived idea and wrote the manuscript. The author confirms being the sole contributor of this work and has approved it for publication.

## Funding

Research in Palli laboratory is supported by NIH (GM070559 and AI163561), NSF (IIP-1821936) and USDA (2019-67013-29351 and 2353057000).

## Conflict of interest

The author declares that the research was conducted in the absence of any commercial or financial relationships that could be construed as a potential conflict of interest.

## Publisher’s note

All claims expressed in this article are solely those of the authors and do not necessarily represent those of their affiliated organizations, or those of the publisher, the editors and the reviewers. Any product that may be evaluated in this article, or claim that may be made by its manufacturer, is not guaranteed or endorsed by the publisher.
